# Effect of Healing Time on Bone-Implant Contact of Orthodontic Micro-Implants: A Histologic Study

**DOI:** 10.1155/2014/179037

**Published:** 2014-04-06

**Authors:** Barat Ali Ramazanzadeh, Kazem Fatemi, Mahboobe Dehghani, Nooshin Mohtasham, Arezoo Jahanbin, Hamed Sadeghian

**Affiliations:** ^1^Dental Research Center, School of Dentistry, Mashhad University of Medical Sciences, Mashhad, Iran; ^2^Oral & Maxillofacial Diseases Research Center, School of Dentistry, Mashhad University of Medical Sciences, Mashhad, Iran; ^3^Department of General Pathology, Faculty of Medicine, Mashhad University of Medical Sciences, Mashhad, Iran

## Abstract

*Objectives*. This study aimed to evaluate the effect of immediate and delayed loading of orthodontic micro-implants on bone-implant contact. *Materials and Methods*. Sixty four micro-implants were implanted in dog's jaw bone. The micro-implants were divided into loaded and unloaded (control) groups. The control group had two subgroups: four and eight weeks being implanted. The loaded group had two subgroups of immediate loading and delayed (after four weeks healing) loading. Loaded samples were subjected to 200g load for four weeks. After sacrificing the animals micro-implants and surrounding tissues were observed histologically. Bone-implant contact ratios (BIC) were calculated and different groups' results were compared by three-way ANOVA. *Results*. Mean survival rate was 96.7% in general. Survival rates were 96.7%, 94.4% and 100% for control, immediate and delayed loaded groups, respectively. BIC values were not significantly different in loaded and control groups, immediate and delayed loading groups, and pressure and tension sides. Mandibular micro-implants had significantly higher BIC than maxillary ones in immediate loading, 4-weeks control, and 8-weeks control groups (*P* = 0.021, *P* = 0.009, *P* = 0.003, resp.). *Conclusion* Immediate or delayed loading of micro-implants in dog did not cause significant difference in Bone-implant contact which could be concluded that healing time had not significant effect on micro-implant stability.

## 1. Introduction

Anchorage is an important factor in achieving optimum results in fixed orthodontic treatment. The success of conventional anchorage reinforcement techniques depends on patients' cooperation; however, some undesirable tooth movements may occur even in the best situations. Using implants for anchorage reinforcement could be helpful in solving these problems [1, 2]. Dental implants are large in size and their surgical procedures and space required make them not preferred choice for orthodontic anchorage. Gradually smaller and simpler implants like onplant, mini-implant, and microimplant were introduced which need to have their own researches.

For orthodontic uses, some fibrous tissue formation in bone-implant interface would be suitable because it facilitates implant removal at the end of treatment. However abundance of fibrous tissue can cause implant mobility. On the other hand the effect of starting time of loading on bone-implant interface is not clear. Considering the above, stability of immediate loaded implants has been subject to serious investigations in recent years [3–5]. In this regard, some histological studies have reported success with immediate loads on dental implants when factors such as primary stability and spilinting between implants are considered [6–8]. On the other hand, microimplants are different from dental implants in size, shape, surface features, and their response to adjacent bones. In addition as applied forces to dental implants and microimplants are also different, it is possible that the required BIC (Bone-Implant Contact) for orthodontic implants would be different from the dental implants.

Many investigations in microimplant issue are on success rate, stability, and removal torque; histological studies are less [9–15]. Woods et al. inserted miniscrews in dog maxilla and mandible and applied 25 and 50 g force after different healing times and evaluated BIC [16]. According to them 25–50 g orthodontic force could be applied immediately. Yano et al. inserted straight and tapered screws in rabbit tibia and assessed BIC after immediate and delay orthodontic loading [17]. They concluded that tapered screws could be loaded immediately but in straight types 6-week healing time should be considered. Deguchi et al. applied orthodontic force to small screws in beagle [18]. According to BIC results they stated that screws could be loaded after 3-week healing time. Zhao et al. evaluated osseointegration of microscrews with *µ*CT imaging system [19]. They concluded that immediate loading may damage stability of microscrews and having a 3-week healing period is more reliable. According to Zhang et al. BIC of orthodontic microscrews significantly increases with healing time [20]. They recommended a 4-week healing time before orthodontic loading. In sum some investigators have recommended that orthodontic forces should be applied to microimplants after a short healing period [18–20]. On the other hand some concluded that time of healing had no significant effect on bone-implant contact [16, 17, 21–23]. However, literatures have not provided clear answer to the question of appropriate time of microimplants healing time. Ensuring from the appropriate time of microimplant loading is an important item for orthodontists. Considering the advantages of microimplant as orthodontic anchorage device, solving the above uncertainties seems to be useful. Apparently further histological studies of bone response to loaded microimplants are required.

The aim of this animal histological research was to evaluate bone-implant contact and survival rate of microimplants after immediate versus delayed orthodontic loading to determine desirable healing time of microimplants.

## 2. Materials and Methods

This interventional animal study was done in animal laboratory of dental research center of Mashhad University of Medical Sciences, Mashhad, Iran. We used four Iranian male dogs. Their mean age was 3 ± 1 year old and mean weight was 30 ± 5 kg. The research protocol was approved by the Ethics Committee and all procedures were done based on animal care guidelines. The animals were selected after three-week quarantine. Inclusion criteria were healthy teeth, not inflamed periodontal tissue, and absence of pathological lesions in the jaws based on the periapical radiography. Then seven-step vaccinations were carried out. All surgeries were performed under sterile conditions in a veterinary operating room and aseptically under general anesthesia.

Sixty-four tapered small head titanium microimplants (Abso Anchor, Dentos Inc., Daegu, Korea; length 6 mm, head diameter 1.4 mm, and tail diameter 1.3 mm) were inserted in the animals' upper and lower jaws. In each animal 16 microimplants (eight assigned loaded and eight unloaded) were placed. Two loaded experimental and two unloaded control microimplants were placed in each quadrant, so 4 microimplants were implanted in each side of maxilla and mandible.

At first, general anesthesia was induced by an intramuscular injection of Xylazine 2% (8 mg/kg) and after 5 ± 2 minutes by intramuscular injection of Ketamine 10% (16 mg/kg).

**Figure 1:**
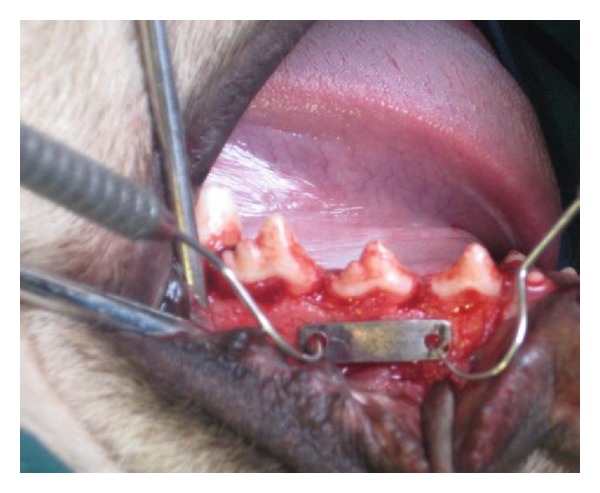
Determination of microimplant position using metallic index.

**Figure 2:**
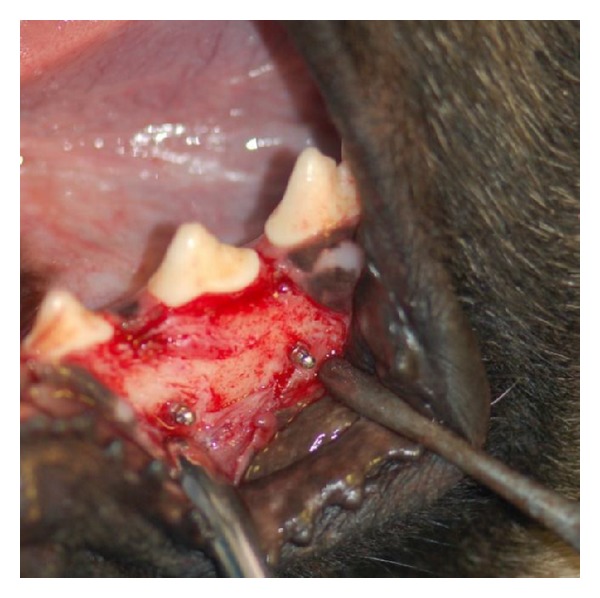
Inserted microimplants.

**Figure 3:**
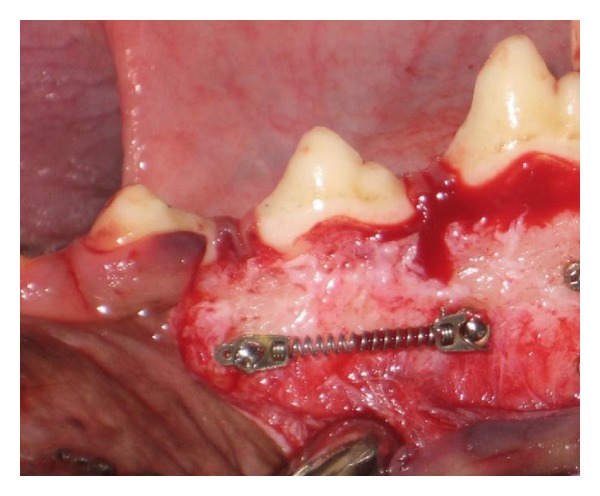
Application of NiTi coil spring for loading.

Local anesthesia (2% lidocaine with 1 : 100.000 epinephrine) was administered via regional infiltration. Then sulcular incisions in the buccal surface of the canine were done to the first molar teeth and periosteal buccal flaps were released using vertical incisions. Using a metallic index, two holes were drilled in the right and left sides of the second premolars, each of 12 mm in distance, as the loaded microimplants were placed in them (Figure 1). Microimplants were inserted perpendicularly in the interradicular bone with a 10 mm distance from the alveolar crest (Figure 2). Unloaded controls were placed near them by at least 7 mm distance on the same day as the experimental microimplants were placed. Using a constant force of 200 g, the effect of delayed (after four-week healing) versus immediate loading was tested in two jaws. The paired experimental groups were perpendicularly activated against each other at the appropriate times with NiTi coil springs with a cross-section diameter of 0.9 mm for constant standardized orthodontic loads of 200 g (Figure 3).

Two dogs, with immediate loading application, were put down within four weeks and the other two dogs, with delayed loading, were killed eight weeks after the first surgery with the vital perfusion method. The maxilla and mandibles were resected en bloc and stored in 10% formalin for 10–15 days prior to sectioning for histological examination. The bone samples were decalcified using 10% of nitric acid for three weeks. After that, microimplants were turned counterclockwise and removed from the bones. The specimens were then mounted in paraffin blocks and cut in slices of approximately 5 µm in direction of microimplants hole long axes as well as loading direction. Then, the slices were put on slides and stained with hematoxylin and eosin (H&E).

**Figure 4:**
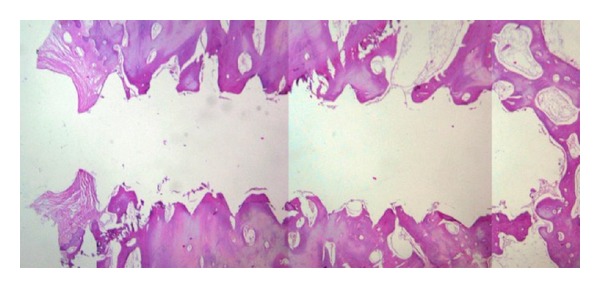
Microscopic view of tissue around a microimplant hole.

For histological assessment an Olympus DP-12 camera mounted on an Olympus BX-41 light microscope was used to digitize each sample at the magnification of 40x (Figure 4). Digitized images were saved as TIFF files and evaluated as histomorphometrical, using the Scion Image Corporation software (Versions 4. 0. 3. 2. Scion) to determine the amount of BIC. The circumference of the microimplants was first traced and recorded using a digital optic pen (Genius G-pen 560) and the BIC was later traced and recorded. The percentage of BIC was calculated as total BIC (microimplant surface length in contact with osseous tissue) divided by total circumference of microimplant ×100. The double blind principle was considered in BIC measuring and analysis.

Data analyses were done using SPSS (V.10). Because of three different variables (maxilla or mandible, experimental or control, and 4 weeks or 8 weeks) we used 3-way ANOVA. Also independent *t*-test was used for statistical comparison. Comparison between tension and compression surfaces was done using paired *t*-test. The significance level was set at 5 percent.

## 3. Results

Among the 64 experimental and control microimplants, only two implants from a single animal showed mobility; one was due to four weeks of control in the mandible and the other was for an immediate loading in the same jaw. The mobile implants were confined to the right quadrant of the lower jaw, so the general survival rate was 96.7%. The general survival rate for the experimental group (*n* = 32) and the control group (*n* = 32) was also 96.7%. The survival rate was 100% *n* = 16 and 94.4% *n* = 16 for delayed loading and immediate loading groups, respectively, and one microimplant was lost in this group.

In some specimens, the histological evaluation showed that microimplants perforated the adjacent roots, so, despite good clinical stability, the mentioned specimens were excluded from the study. In addition, some cases were excluded from the histological study because of the poor quality of their slices; so in all, 51 cases were included for statistical analysis.

According to Table 1, there were significant differences in BIC between the maxilla and the mandible for immediate loading group, 4-week control group, and 8-week control group.  Table 2 shows there were no significant differences in BIC between immediate versus delayed loading and between 4-week control and 8-week control in each jaw.

We also compared the BIC in pressure and tension surfaces. The results showed that there were no significant differences between groups (Table 3). Histological analysis showed that the bone around the mandibular microimplants was often cortical (96.4%) with a little spongious bone (3.6%); however, for the maxilla, the spongious bone was 56.5% and the cortical bone was 43.5%. Other tissues around the microimplants included fibrous tissue, vessels, and bone marrow.

According to Table 4, in all groups, fibrous tissue was more formed in the cervical third of the microimplant circumference. However, the mean fibrous tissue was 4.27% and 10.21% for the middle third and the apical third, respectively. In other words, the least BIC was due to the cervical third and the most BIC was due to the apical two-thirds.

## 4. Discussion

Orthodontic treatment dynamics are related to anchorage stability and absolute anchorage is desirable for decreasing duration of treatment and achieving treatment goals. In this study, we evaluated the influence of 200 g immediate and delayed load on microimplants in an animal model. There were no significant differences in bone-implant contact between immediate and delayed loading and between loaded and unloaded samples.

Microimplant loss before loading has been reported in some articles [16, 18, 22]. Freire et al. reported a survival rate of 66.6%; however, that was more than the survival rate of this study (96.7%) [24]. The difference may be due to surgical errors, hard nutrition, soft tissue inflammation, and differences in animals' races. In Freire et al. study all of the lost microimplants were due to the same animal which was similar to the present study. Individual differences may cause different responses to similar stimulus; however, anatomic variables such as the value of interdental bone may be responsible for different survival rates in different individuals. Freudenthaler and some others reported peri-implant soft tissue inflammation as a major cause of microimplant loss [18, 25–27]. Similarly in our study histological evaluation of the lost microimplants revealed inflammation in peri-implant areas, which closely correlates with the Woods et al. study [16]. The fact that only two microimplants which were related to both control and experimental groups were lost show that primary factor causing microimplant loss is not orthodontic load application. The high survival rate of immediate loaded microimplants (94.4%) shows microimplants can provide reliable anchorage even under immediate loading.

The range of BIC in this study was 62.69%–88.22%. In previous histological studies, the range of reported BICs is wide and varied [17, 18, 22–24, 28]. In Deguchi et al. maximum BIC of miniscrews was 40% which is lower than our results [18]. The difference may be due to different technical methods like the thickness of tissue slices, method of microimplant removal, and method and software for BIC measuring. In current study tissue slices thickness was 5 µm, while in Deguchi study the tissue slices were prepared in 100 µm thickness. More slice thickness decreases the accuracy. In Ma et al. study the reported range of BICs was 59–62% which is approximately near to the present study [23]. Çehreli and Arman-Özrpc reported the BIC of self-tapping microimplants at the time of insertion as 80.73% [29]. However the minimum BIC for clinical success in orthodontics has not been clearly described yet. In this study, the microimplants with 40.82% of BIC were fully stable. Woods et al. showed that even 2.2% of BIC can create good stability [16]. Deguchi et al. reported some samples with 5% of BIC, which had been well loaded with orthodontic forces [18]. In spite of that, because of the different or not explained BIC measuring methods, comparing the results of different studies is difficult. Generally it could be concluded that good stability could be achieved with minimum BIC.

The maximum BIC was seen in the apical two-thirds, while the minimum BIC was detected in the cervical third. This result coincides with the findings by Woods et al. and Luzi et al. studies [16, 22]. As we know, cortical bone is more prevalent in the cervical region than in the other areas and this fact may be in contrast with our results. The cause of this contradiction may be due to contact time of the cervical third by the drill, greater possibility of trauma to the cervical third than other areas of microimplants, and inflammatory reactions in soft tissues of microimplant heads.

According to Table 1 BIC of microimplants, both in experimental and the control groups, increased over time; however, the value was not statistically significant. These findings are in line with some other investigations. Freire et al. showed that the BIC in samples with 12-week healing was more than the ones with 1-week healing [24]. Also Luzi et al. showed from 4 weeks to 12 weeks BIC progressively increases [22].

In regard to the immediate versus delayed loading, BIC of immediate loaded microimplants was just slightly less than delayed loaded ones and the differences were not statistically significant. According to Woods et al. BIC of immediate versus delayed loaded miniscrews was not significantly different which is consistent with us [16]. Their results showed immediate loading in the range of 25–50 g force is safe. Also Luzi et al. stated that immediate loading with light forces (50 g) did not negatively affect bone healing [22]. Usually clinical orthodontic loads are higher. So we tested 200 g force which is rather heavy. Results showed microimplants could be loaded immediately even under heavy orthodontic loads. Freire et al. showed BIC in samples with 3-week healing time was more than the immediate loading ones [24]. They used mini-implants with diameter and structure more near to dental implants in comparison with currently used microimplants. Maybe some surface differences of their samples significantly enhanced osseointegration with time. Despite that clinical stability for both immediately and delayed loading groups was acceptable. Deguchi et al. recommended 3-week healing time before loading because they did not find significant differences between early (after 3-week healing) versus delayed loading [18]. The different result with us may be due to that in Deguchi study effect of immediate loading was not assessed and the least tested healing time was 3 weeks which showed successful result. Ma et al. showed success of immediate loading of microimplants in dog [23]. However they implanted only in mandibular bone which has dense cortical bone and generalizing the results to maxillary bone with less density should be with caution. Anyway this comparison was done in our study and the results of immediate loading in both jaws were satisfying. Zhao et al. recommended a minimum of 3-week healing time before loading because in their study osseointegration was progressively increased by the end of the third weeks [19]. However microscopic computerized tomography was the method of osseointegration assessment in that study which was different from current histological study. It should be mentioned that, despite the statistically significant more bone-implant contact of 3-week delayed loaded microscrews in Zhao study, survival rate of all immediate and delayed loaded samples was high and similar. Maybe 3-week waiting before loading has not clinical significance. In our study BICs in pressure sides of microimplants were not significantly different from tension sides. Apparently similar to the timing of force application, type of force as compressive or tensile has not important effect on bone response. This finding is in agreement with Woods et al. [16].

In contrast to the dental implants, there is no need for long healing duration for the microimplants due to the fact that it is not necessary to have osseointegration for the device that should be removed at the end of treatment. Considering this fact, it may be suggested that immediate loading is better than the delayed one. According to the results the BIC in immediate loading was equal or a little smaller than delayed loading, but clinical stability was high enough in both, while lower BIC could facilitate the removal of the microimplants at the end of treatment.

As limitations, the study was animal one because we aimed to make a histologic evaluation. However direct generalization of the findings of an animal study to clinical situations should be with caution. Also in this study the microimplants were removed before histological sections. Nondestructive tests may be better. The orthodontic load was static and the microimplants were not used as anchorage of real orthodontic movements. Clinical studies on the effect of healing time on stability of microimplants are recommended for future.

## 5. Conclusions

As a conclusion of our animal research there were no significant differences in bone-implant contacts between loaded and unloaded microimplants and between immediate and delayed loading. In sum, static orthodontic force application and the time of loading have not significant effect on BIC of microimplants. Within the limits of this study, immediate loading after microimplant insertion could be recommended.

## Figures and Tables

**Table 1:** Comparison bone-implant contact (BIC%) of microimplants in maxillary and mandibular groups.

Group	Maxilla	Mandible	Independent
	Mean (%) ± SD	Mean (%) ± SD	*t*-test
Loaded			
Immediate	62.69 ± 10.6	76.59 ± 9.6	*P* = 0.021*
Delay	67.42 ± 8.3	79.67 ± 14.7	*P* = 0.149

Unloaded			
4 weeks	65.72 ± 15.5	84.71 ± 4.6	*P* = 0.009*
8 weeks	70.43 ± 10.4	88.22 ± 14.04	*P* = 0.003*

*Significant at <0.05.

**Table 2:** Comparison of bone-implant contact (BIC%) between immediate/4 weeks and delayed/8 weeks and between loaded and unloaded (control) groups.

Group	Immediate/4 weeks	Delayed/8 weeks	Independent
	Mean (%) ± SD	Mean (%) ± SD	*t*-test
Maxilla			
Loaded	62.69 ± 10.6	67.42 ± 8.3	*P* = 0.426
Unloaded	65.72 ± 15	70.43 ± 10.4	*P* = 0.542
Independent *t*-test	*P* = 0.663	*P* = 0.623	

Mandible			
Loaded	76.59 ± 9.6	79.67 ± 14.7	*P* = 0.669
Unloaded	84.71 ± 4.6	88.22 ± 14.0	*P* = 0.180
Independent *t*-test	*P* = 0.202	*P* = 0.069	

**Table 3:** Comparison of bone-implant contact (BIC%) between tension and pressure surfaces of loaded groups.

Group	Mean (%) ± SD	Paired *t*-test
Maxilla		
Immediate		
Pressure	62.51 ± 15.5	*P* = 0.97
Tension	62.87 ± 17.8	
Delayed		
Pressure	71.08 ± 14.7	*P* = 0.377
Tension	63.76 ± 8.5	

Mandible		
Immediate		
Pressure	75.23 ± 9.3	*P* = 0.173
Tension	77.95 ± 10.4	
Delayed		
Pressure	83.10 ± 14.5	*P* = 0.141
Tension	76.23 ± 16.0	

**Table 4:** Distribution of soft tissue (%) around the microimplants.

Group	Cervical third	Middle third	Apical third
	Mean (%) ± SD	Mean (%) ± SD	Mean (%) ± SD
Maxilla			
Loaded			
Immediate	78.62 ± 31.2	4.75 ± 11.5	16.62 ± 26.5
Delayed	100 ± 0	0	0
Unloaded			
4 weeks	72.28 ± 24.4	9.71 ± 13.8	18 ± 20.4
8 weeks	100 ± 0	0	0

Mandible			
Loaded			
Immediate	64.28 ± 22.9	7.14 ± 12.1	28.57 ± 20.9
Delayed	88.00 ± 26.83	6 ± 13.14	6 ± 13.4
Unloaded			
4 weeks	90.86 ± 17	4.57 ± 8.5	4.57 ± 8.5
8 weeks	100 ± 0	0	0

Total	85.51 ± 23.1	4.27 ± 9.7	10.21 ± 18.1
